# Liquid Chromatographic Tandem Mass Spectrometric (LC-MS/MS) Determination of Perfluorooctane Sulfonate (PFOS) and Perfluorooctanoic Acid (PFOA) in the Yolk of Poultry Eggs in Malaysia

**DOI:** 10.3390/molecules25102335

**Published:** 2020-05-16

**Authors:** Atiqah Tahziz, Didi Erwandi Mohamad Haron, Mohd Yusmaidie Aziz

**Affiliations:** 1 Integrative Medicine Cluster, Advanced Medical and Dental Institute, Universiti Sains Malaysia, Kepala Batas, Penang 13200, Malaysia; atiqahta@student.usm.my; 2 Shimadzu-UM Centre of Xenobiotic Studies, Faculty of Medicine, University of Malaya, Kuala Lumpur 50603, Malaysia; didi27@um.edu.my

**Keywords:** perfluorooctane sulfonate (PFOS), perfluorooctanoic acid (PFOA), poultry eggs, liquid chromatography tandem mass spectrometry (LC-MS/MS)

## Abstract

Perfluorooctane sulfonate (PFOS) and perfluorooctanoic acid (PFOA) are widely used in products, and are known for their water and grease repellent properties. The persistence nature and potential toxicity of these substances have raised substantial concerns about health effects. Regarding humans, food consumption has reportedly been a significant source of exposure for both compounds. Hence, this study was performed to develop and validate an analytical method for PFOS and PFOA in egg yolks using liquid chromatographic tandem mass spectrometry (LC-MS/MS) followed by the determination of concentration of both compounds in the yolk of poultry eggs in Malaysia. A total of 47 poultry egg yolk samples were extracted by a simple protein precipitation technique using acetonitrile. The analytical method was developed using LC-MS/MS and validated based on the Food and Drug Administration (FDA)’s Bioanalytical Method Validation guidelines. The results revealed that PFOS was quantitatively detected in six samples, with the concentration range between 0.5 and 1.01 ng g^−1^. Among these, five samples were from home-produced chicken eggs, and one sample was from a quail egg. The levels of PFOA in all samples were below the quantifiable limit (<0.1 ng g^−1^). This indicated that the contamination of PFCs in poultry eggs were mostly attributed to the nature of free foraging animals, which had direct contact with the contaminants in soil and feed. In conclusion, a fast and robust analytical method for analyzing PFOS and PFOA in egg yolk samples using LC-MS/MS was successfully developed and validated. The presence of these emerging contaminants in this study signified widespread pollution in the environment.

## 1. Introduction

Perfluorinated compounds (PFCs) are manmade organic chemicals that are widely used as surfactants and surface protectors in many products; they are known for their unique characteristics, particularly their grease, stain, and water repellent properties [1]. PFCs are used, for example, as basic materials in automobiles, aviation, chemical industries, textiles, electronics, as well as in semi-conductors. These chemicals have high chemical and biological stability, mainly attributed to the chemical structures, displayed by the strength of the bond between carbon and fluorine atoms [2]. Considering their persistence, PFCs are found, ubiquitously, in the environment, and have bioaccumulated in the food chain [3].

**Figure 1:**
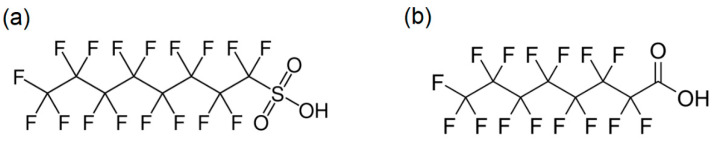
Structure of (**a**) Perfluorooctane sulfonate (PFOS) and (**b**) Perfluorooctanoic acid (PFOA).

The two most discussed PFCs are perfluorooctane sulfonate (PFOS) and perfluorooctanoic acid (PFOA); they are globally identified and have recently raised international concern. Both PFOS and PFOA are long chain perfluoroalkyl substances (eight carbon chains) with the following chemical formulas: C_8_F_17_SO_3_^−^ and C_8_F_15_COO- [4] (Figure 1). Unlike other classical lipophilic persistent pollutants, such as dioxin and polychlorinated biphenyls, both PFOS and PFOA do not typically accumulate in lipids, but rather in body compartments with high protein content [5]. Toxicology studies in animals and biomonitoring data from occupationally exposed workers in PFC industries, have shown the potential of these compounds causing health implications associated with liver toxicity, immunological and endocrine disruption, development toxicity, as well as cancer [6].

The main pathway for human exposure to PFCs would be from dietary intake, sourced from contaminated food and/or water [7]. The intake of fish and marine mammals were reportedly the main contributors to dietary exposure of PFCs, apart from their presence in various other food sources [8]. In recent years, the presence of PFCs in chicken eggs was reported [2,9]. Chicken eggs are identified as a common source of protein intake in human diets. Some of these eggs are collected from chickens that are reared non-commercially, free-range, and mainly feed from pecking worms or small insects from the soil. These chickens, which are exposed to the external environment, may have their products (e.g., eggs) become contaminated with pollutants, such as PFCs. To date, not much information is available concerning human exposure to PFCs from dietary intake in Malaysia.

There has been increasing demand towards analysis of PFCs in environmental and biological mediums. The challenges in determining the presence and distribution of PFCs are mostly due to the occurrence of these compounds in different phases and the complexity of biological matrices, especially in biota [10]. In the past, researchers have faced many analytical challenges due to the relatively low concentrations of PFCs in most samples, the scarcity of pure authentic standards and internal standards, as well as the difficulties in sample extraction and preparation techniques [11]. The use of highly sensitive instruments, such as liquid chromatography tandem mass spectrometry (LC-MS/MS), has enable the measurement of these compounds (low pg/mL (ppt)), which has been beneficial to the research in this field. In addition, the use of ultra-high-performance liquid chromatography (UHPLC) has enabled fast separation and high-resolution analysis, providing better separation of PFCs in complex samples, such as in food matrices [10]. Nevertheless, there are still a lot of difficulties in developing and performing analysis of these emerging persistent compounds. Poor recoveries, inter-laboratory differences, matrix complexities, and the presence of structural isomers in biological samples are some of the many challenges discussed [12,13]. In biota and food analysis, the presence of interfering components, such as lipid, proteins, organic matter, and pigments, can lead to ion suppression of the target compounds, which would highly affect the sensitivity of the analysis [13]. Furthermore, the extraction and sample preparation of PFCs are mostly time consuming and costly. Most laboratories have to utilize extensive clean-up techniques, such as solid phase extraction (SPE), in order to achieve high recovery of the target compounds. However, despite all of the presented challenges, many efforts have progressively been made to improve the quality of analytical work, in order to bring about highly reliable and sensitive results. Thus, this study aims to develop an analytical technique for the quantitation of two common PFCs (PFOS and PFOA) in poultry eggs, using LC-MS/MS, by adapting simple procedures to overcome some of the analytical challenges. This developed and validated method, for the first time, is used to investigate the PFOS and PFOA contamination in poultry eggs (chicken, duck, and quail) in Malaysia.

## 2. Results

### 2.1. Selection of Deprotonated Ions

**Figure 2:**
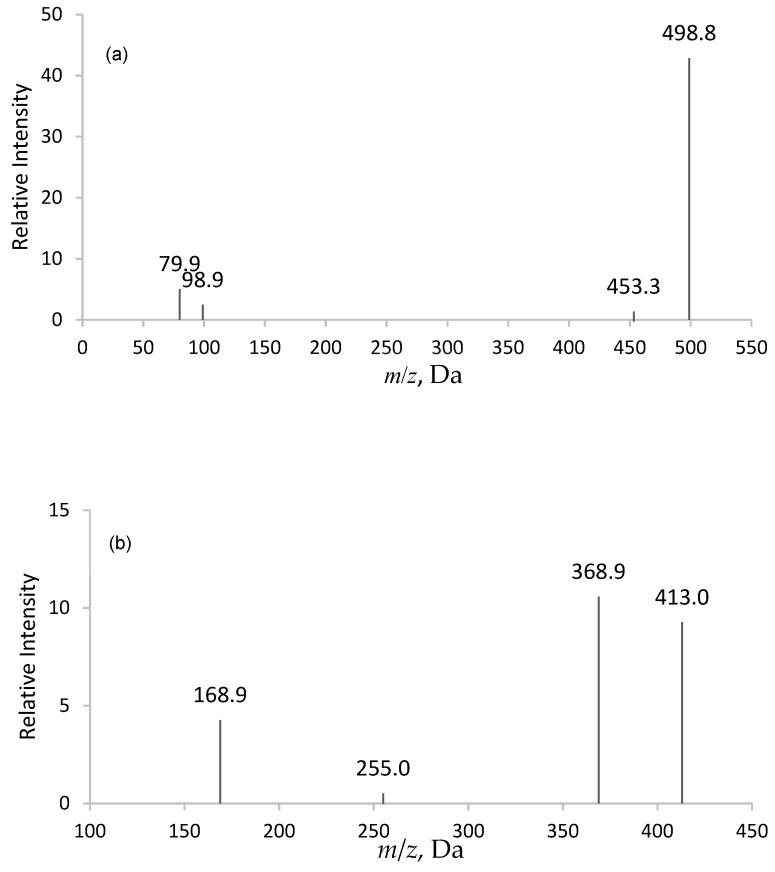
Spectrum for (**a**) Perfluorooctane Sulfonate (PFOS) and (**b**) Perfluorooctanoic Acid (PFOA).

The product ion spectrums of PFOS and PFOA are shown in the diagram below (Figure 2). Following the infusion of analytes into the mass spectrometer, the mass spectrum of PFOS showed a molecular ion [M − H]^−^ at 498.800, with major fragments of *m*/*z* 98.900 and 79.900. In order to avoid the interference from sodium taurodeoxycholate hydrate (TDCA), 98.900 was selected as the primary ion transition for quantitation. As for PFOA, the mass spectrum showed a deprotonated molecular ion [M − H]^−^ at 413.000, with major fragments of *m*/*z* 368.900 and 168.900. The primary ion transition selected for PFOA quantitation was 413.000 → 368.900. The mass transition of 502.668 → 80.00 and 416.752 → 371.800 was selected for both ^13^C_4_PFOS and ^13^C_4_PFOA, respectively.

### 2.2. Assay Specificity and Selectivity

**Figure 3:**
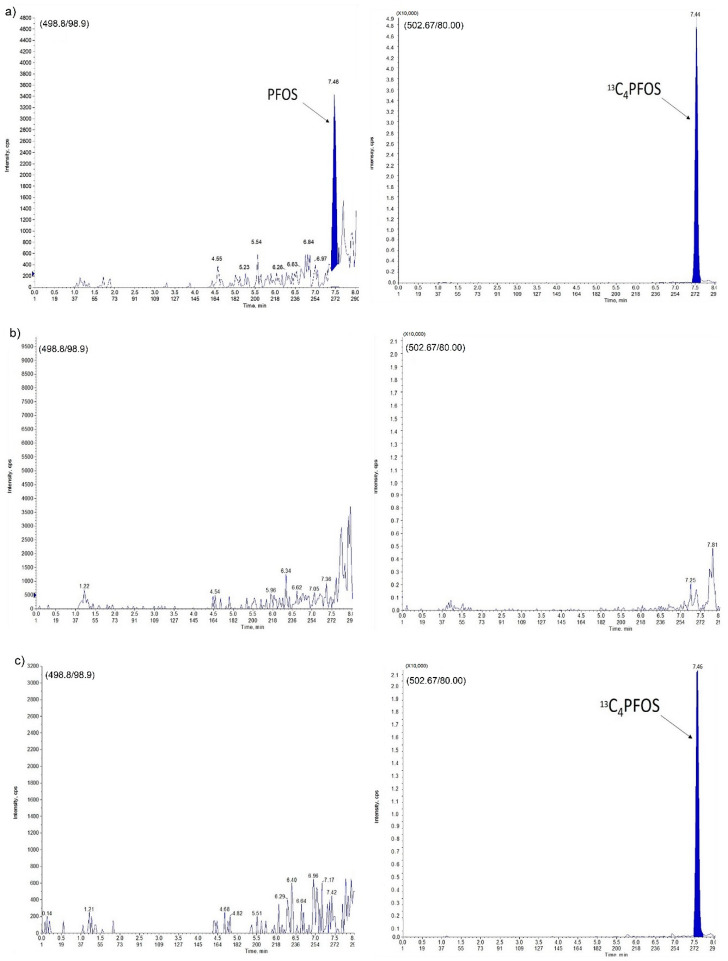
Perfluorooctane Sulfonate (PFOS) in egg yolk sample: (**a**) LLOQ; (**b**) double blank sample; (**c**) zero blank sample.

**Figure 4:**
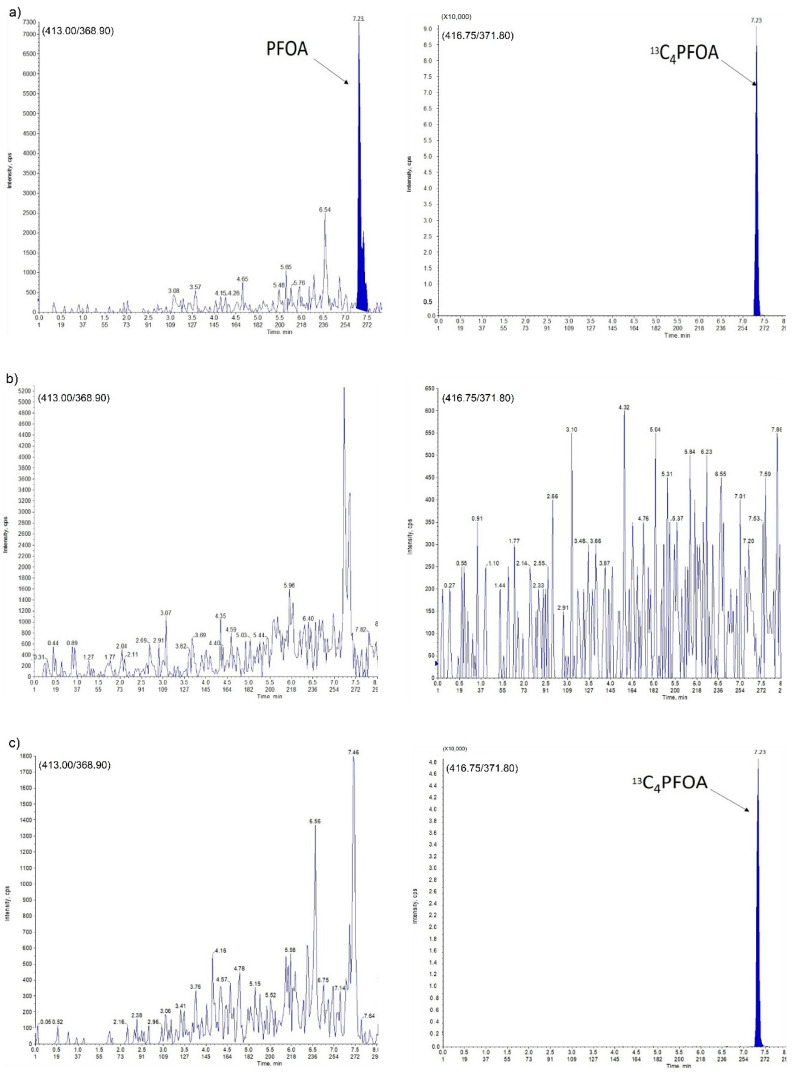
Perfluorooctanoic Acid (PFOA) in egg yolk sample: (**a**) LLOQ; (**b**) double blank sample; (**c**) zero blank sample.

The assay specificity and selectivity were evaluated by analyzing four individual samples of blank egg yolk (containing neither analyte nor internal standard (IS)), to exclude any endogenous co-eluting interference in the peak region of both analyte and IS. The results showed that none of the double blank samples had peaks co-eluting with an area higher than 15% of lower limit of quantitation (LLOQ). In addition, no peaks higher than 5% of the IS peak area were detected in the blank yolk. The chromatograms are shown in Figure 3 and Figure 4. The analyte retention times for PFOS and PFOA were 7.46 and 7.23 min, respectively.

### 2.3. Calibration Curve and Sensitivity

The quantitation for PFOS was evaluated using seven point calibration curves, in the range from 0.5 ng g^−1^ to 20.0 ng g^−1^. For PFOA, the quantitation was evaluated using nine point calibration curves, covering the range from 0.1 ng g^−1^ to 20.0 ng g^−1^. The data were fit into a quadratic, 1/x-weighted regression equation. The coefficient of determination (r^2^) of the calibration curve for both compounds from inter-day analyses was found to be greater than 0.99.

### 2.4. Precision and Accuracy

The inaccuracy for both compounds (PFOS and PFOA) at all quality control (QC) concentration levels were within ±15%. The relative standard deviation (RSD), expressed as coefficient of variance (% CV) were determined for both inter- and intra-assay run. All results of inter- and intra- assay reproducibility falls within the range of <15% CV (QC low, mid and high) and within the range of <20% CV for LLOQ, as shown in Table 1.

### 2.5. Stability

The autosampler stability for both PFOS and PFOA was determined by storing the QC-low and QC-high concentration samples, up to 24 h in the autosampler (*n* = 5). The accuracy for PFOS falls in the range of 94.35% to 108.5%, while the precision (% CV) lies in the range of 1.88% to 2.21%. The accuracy for PFOA falls in the range of 93.51% to 99.10%, while the precision (% CV) lies in the range of 0.02% to 4.29%. Table 2 summarizes autosampler stability results for both PFOS and PFOA.

### 2.6. Concentration of Perfluorinated Compounds in Yolk Samples

The concentrations of PFOS and PFOA from 47 egg yolk samples sourced from chicken, duck, and quail were tabulated in Table 3. Thirty samples of commercially produced eggs contained unquantifiable concentrations of PFOS (<0.50 ng g^−1^) and PFOA (<0.10 ng g^−1^), respectively. Out of 47 egg yolk samples analyzed, six were found to contain PFOS at or above the level of LLOQ (0.5 ng g^−1^). In these six egg yolk samples, five were from home produced chicken eggs, while the remaining one was from quail eggs. None of the egg yolk samples screened contained PFOA at quantifiable levels.

The distribution of analyte in samples was analyzed using SPSS version 24. It was observed that the concentration of PFOS and PFOA in all of the egg samples analyzed did not follow the normal distribution; hence, descriptive data are presented in median and interquartile ranges. The concentration of PFOS in all of the samples analyzed fell in the range of <0.50 ng g^−1^ to 1.01 ng g^−1^; in PFOA, all of the results were <0.10 ng g^−1^.

## 3. Discussion

### 3.1. Sample Preparation for LC-MS/MS Analysis

Determination of both PFOS and PFOA require a very sensitive method, whereby trace amounts of the compounds are present in the environment or food matrices. Thus, LC-MS/MS instrumentation was chosen for the analysis. The initial part of this study involves the optimization of the sample preparation procedure. Several combinations of clean-up and extraction procedures were attempted, which included alkaline digestion, simple protein precipitation, ultrasonication, as well as solid phase extraction (SPE). In the first attempt, alkaline digestion coupled with solid phase extraction was carried out, with reference to the method described by So et al. [14]. The alkaline digestion step was performed in order to release perfluorinated compounds (PFCs) from proteins, since PFCs are known to show specific protein-binding properties. It was suggested that this step aids in obtaining reliable recovery of PFCs in biological samples. The procedure was carried out by adding 2 mL of 200 mM sodium hydroxide in methanol to every sample, and the mixture was shaken for 1 h (250 g). The methanol extract was then subjected to further clean-up by solid phase extraction. The SPE procedure was then evaluated using two different cartridges in order to compare the recovery capacity of each. These two cartridges were Oasis^®^ WAX (3 cc, 60 mg, Waters, Millford, MA, USA) and Oasis^®^ HLB (6 cc, 200 mg, Waters Millford, MA, USA). Oasis^®^ WAX is a mixed-mode weak anion-exchange cartridge, used for the purpose of retaining and releasing strong acids, and the polymer is stable in organic solvents. On the other hand, Oasis^®^ HLB contain polymer with a unique hydrophilic–lipophilic balance, and the sorbent, is ideal for acidic, basic, and neutral analytes. Following the clean-up step by solid phase extraction (SPE), the eluent was dried under a gentle stream of nitrogen gas. The dried eluent was reconstituted with methanol, prior to analysis by LC-MS/MS. The analyte recovery of this procedure, evaluated using the two different cartridges, showed a relatively unsatisfactory result. For the workout using Oasis^®^ WAX cartridge, the mean recovery achieved for analyte-spiked matrix (at 5 ng g^−1^) was only 30%. The evaluation using Oasis^®^ HLB also showed similar results, in which the recovery obtained for analyte-spiked matrix (5 ng g^−1^) was only 32%. This procedure was further optimized using hard-boiled egg yolk, based on the method described by Zafeiraki et al. [2]. According to the method, boiling the egg yolk improves the sensitivity of the extraction technique. The sample was subjected for the similar clean-up and extraction procedure, with an additional step of ultrasonication (20 min) after alkaline digestion. However, the attempt was to no avail, as the recovery achieved was still unsatisfactory (55–60%). The extraction procedure was finally evaluated using the simplest approach—a simple protein precipitation technique. This procedure was performed based on the method described by Malinsky, Jacoby, and Reagen [15], with a few modifications. Acetonitrile was selected as the extraction solvent, as this is one of the ideal solvents that can precipitate proteins, as reported by a previous method [15,16,17]. In addition, the evaporation rate of acetonitrile is greater when compared to methanol (as methanol is more polar than acetonitrile); thus, this resulted in a faster drying process under the nitrogen stream. Principally, the addition of acetonitrile to the egg yolk samples resulted in the aggregation of proteins, in which, following centrifugation, settled as a pellet at the bottom of the sample vial. This pellet was easily removed; thus, resulting in fast and easy separation of proteins and small molecules. Following drying under a gentle stream of nitrogen gas, the dry residue was reconstituted using methanol, and filtered using a syringe filter (Phenex reverse cellulose syringe filter, pore size 0.2 µm). The analyte recovery achieved through simple protein precipitation showed good and reproductive results. The mean recovery for analyte-spiked matrix, evaluated in quality control (QC) samples, was in the range of 84% to 102% for both perfluorooctane sulfonate (PFOS) and perfluorooctanoic acid (PFOA). When we compared these results with those obtained after SPE clean-up, the latter showed a significantly lower recovery, mostly due to loss during the analyte washing steps or insufficient elution. Hence, due to the outcome, simple protein precipitation was decided as the method of choice for the extraction technique, due to the improved recovery, as well as reduced analytical costs and processing time. However, it was also noted that the decision to utilize this simple technique would result in certain drawbacks during the instrumental analysis. The simplicity of the technique results in less clean samples as other matrix components (other small contaminating molecules) are not efficiently removed and, hence, may contribute to matrix effects in the samples. Nevertheless, this factor can be tolerated, considering the good and reproducible recovery (within European Union (EU) recommendation 21010/161/EU) for both PFOS and PFOA in the analyzed samples (European Commission, 2010) [18]. Furthermore, the straightforward, simple protein precipitation technique led to fast and high throughput analysis on the overall sample preparation, compared to other methods that have been evaluated.

### 3.2. LC-MS/MS Method Validation

All procedures conducted during method validation were performed using blank egg yolk samples spiked with native PFC standard mixtures, as well as internal standards (^13^C_4_-PFOS and ^13^C_4_-PFOA). The preparation of samples and standards were performed in polypropylene containers, as it was reported that PFCs can potentially adsorb to glass surfaces [19]. Careful measures were also taken in ensuring that there was no contact with polytetrafluoroethylene (PTFE) containing materials, which can be a source of contamination during the procedural analysis. The stock standards of PFOS and PFOA, as well as the corresponding isotopes, were prepared in 100% methanol. Each batch of extracted samples included a procedural blank to control external contamination throughout the whole analytical process. In the analysis of PFCs, there is potential of method interferences from the sources, such as solvents, reagents, or instrumental hardware. Thus, in the first step, solvent blanks (methanol and acetonitrile) were analyzed to control background interferences from the mobile phase and instrument. The results showed that no traces of PFOS and PFOA were detected. Specificity and selectivity were further evaluated by screening procedural blank samples (blank egg yolk samples, *n* = 4). Target compounds were not detected in any of the double blank samples. The analysis of zero blank samples and samples spiked at the LLOQ level also showed no peaks co-eluting in the respective retention time. The variation of PFC retention times were found to be within ±2.5 s. In order to assess potential response variations that may affect the quantification of samples, a seven point calibration curve for PFOS (0.5 ng g^−1^ to 20 ng g^−1^) and a nine points calibration curve for PFOA (0.1 ng g^−1^ to 20 ng g^−1^) were evaluated. The range of points in the calibration curves were ensured to include the limit of quantitation 1 ng g^−1^, as suggested by The European Food Safety Authority (EFSA) Commission Recommendation 2010/161 [18]. In this study, the data were fitted into a quadratic regression equation, as the relationship between the measured signals and the analyte concentrations was non-linear. This was largely due to the matrix effect of the samples, as we have noted before, attributed by the simple sample preparations (protein precipitation technique). This occurrence was noted in samples analyzed by LC-MS/MS in several other studies [15,20] and, hence, the option of quadratic regression is acceptable. The 1/x weighting factor was opted in the calibration curve in order to give more emphasis to the lower concentrations, as well as to ensure good assay performance. The coefficient of determination (r^2^) for both PFOS and PFOA were greater than 0.99 in all of the calibration curves constructed during the analysis. The developed method had shown satisfactory sensitivity with LLOQ set at 0.5 ng g^−1^ for PFOS, and 0.1 ng g^−1^ for PFOA. These values agree with the standards set by the EFSA Commission Recommendation 2010/161, where they recommend the limit of quantification of 1 ng g^−1^ in monitoring of perfluorinated compounds in foods. Accuracy and precision of the method were evaluated for both intra and inter-day analyses. The RSD (% CV) for intra and inter-day analyses for PFOS and PFOA did not exceed the levels of 15% from QC nominal concentration and was less than 20% for LLOQ. The intra and inter-day evaluation of accuracy for both PFCs, on all QC concentrations, were in ±15% of the calculated values. These reference values were based on guidelines by the United States Food and Drug Administration (USFDA) [10]. In this study, the relative recovery workout was performed by comparing the peak area response in the egg yolk sample fortified with the analytes (PFOS and PFOA) before extraction (pre-spiked samples), with the peak area response in a blank yolk sample that was fortified with the analyte after extraction (post-spiked samples). This method was performed with the idea to consider the matrix effect. The EU recommendation 21010/161/EU has set ideal recovery rates, in the 70% to 120% range, for the quantitative analysis of perfluorinated compounds in food. The extraction of egg yolk samples using simple protein precipitation in the analysis lies within the recommended range, with the results covering the range of 92.8% to 101.5% for PFOS, and 83.9% to 91.1% for PFOA. The precision (% CV) of the recovery at each concentration level was less than 10%. Due to good recovery results, the simple extraction method was applicable throughout the analysis. Stability testing is important to assess the chemical integrity of the analytes. Analyte instability may result in inaccurate analytical estimation (over or under-estimated). It was well known that perfluorinated compounds, such as PFOS and PFOA, are chemically and thermally stable [21]. However, these might be compromised during the processing and storage of the samples. Hence, in this study, the stability of PFOS and PFOA under autosampler condition (±15 °C) was evaluated. Both analytes were found stable, with acceptable accuracy and precision. These showed that following extraction, the egg yolk samples can be analyzed over 24 h, in an autosampler at ±15 °C, without any loss or degradation.

### 3.3. Concentration of Perfluorinated Compounds in Egg Yolk Samples

Another goal of the study is to determine the level of perfluorooctane sulfonate (PFOS) and perfluorooctanoic acid (PFOA) in the yolk of poultry eggs (chicken, duck, and quail) in Malaysia. It is important to evaluate the presence of perfluorinated compounds in eggs, since they serve as sensitive bioindicators of the persistent organic pollutants [22]. It was reported that one of the elimination pathways of pollutants is through deposition in eggs [23], and the study of PFC contamination in eggs reflects the contaminant burden in adult animals [24]. Moreover, eggs are a common source of dietary intake among the Malaysian population. In the present study, 47 egg yolk samples from chickens, duck, and quail were analyzed for the presence of PFOS and PFOA. At this point, this number of samples are sufficient to provide initial data on the presence of PFCs in egg samples, and is relevant with other studies conducted on eggs [2,9]. According to the study by Wang et al. [24], PFCs were primarily detected in the egg yolk, with much lower concentrations detected in the albumen. This was reportedly due to the binding behavior of these compounds, with different types of proteins in the yolk and albumen. Hence, in this study, the analysis was only performed in egg yolks. Between the two PFCs screened in the samples, PFOS was dominantly detected, as PFOA concentration in samples did not reach the quantifiable limit. PFOS was detected in six samples, of which five were home produced chicken eggs, and one was from quail eggs. In chicken eggs, the analysis was done to compare the level of PFOS and PFOA between commercially produced and home-produced sources. The results showed that none of the commercially produced chicken eggs contained any detectable level of PFOS nor PFOA. This finding was found to be consistent with previous study by Zafeiraki et al. [2], whereby the PFAS levels detected in almost all of the commercial egg samples were below quantifiable limit. On the contrary, five yolk samples from home produced sources were found to contain PFOS in the concentration range of 0.50 ng g^−1^ to 1.01 ng g^−1^. The highest concentration (1.01 ng g^−1^) was observed in a sample from Terengganu. However, the location was not known to be near any manufacturing industries, and we assume that the contamination may come from the source of PFC-containing wastes, which contaminated the soil or water source. Other studies showed that PFOS concentration in home produced chicken egg samples from Malaysia (0.50 ng g^−1^ to 1.01 ng g^−1^) are lower compared to those reported from other countries, such as the Netherlands (0.50 ng g^−1^ to 24.8 ng g^−1^) and Greece (0.50 ng g^−1^ to 8.9 ng g^−1^) [2]. PFOA concentrations in all chicken egg samples, which are found to be below LLOQ in this study, does not differ much from findings in other countries (China, Netherlands, Greece, and Spain) which also reported low concentration of PFOA [2,9,25]. The presence of PFOS in home produced chicken eggs can be related to their close contact with the external environment. As discussed earlier, one of the possible exposure pathways for perfluorinated compounds are from ingestion of contaminated food and water. Home produced chicken eggs are obtained from free-range chickens, which mainly catch their feed of worms, insects, or food waste, on the ground. The action of scavenging and scratching the soil might also expose them to ingest a certain amount of soil during feeding. It was clearly known from many studies that soil, apart from water, serves as a significant non-biota environmental medium for the spread of pollutants, mainly of persistent organic pollutants and heavy metals [26,27,28]. In contrast, commercially produced chickens are mainly kept indoors, within the farm, and these chickens are fed commercial feed. Therefore, the livestocks have limited access to the external environment. Some are organic reared chicken; hence, they are fed with good, high-grade natural feed, such as grains, herbs, and fruits. All of these factors, possibly, contribute to the absence of perfluorinated compounds in commercially produced chicken eggs. Compared to chicken eggs, the analysis done on duck and quail eggs showed PFOS and PFOA concentration below the quantifiable limit, except for one quail sample, displaying PFOS concentration of 0.69 ng g^−1^. Assessment of perfluorinated compounds in food has been conducted in many other countries, such as China, Netherlands, Greece, Canada, United Kingdom, and Spain [2,14,29,30,31]. In Malaysia, monitoring of perfluorinated compounds are more focused on non-biota environmental pollution, such as in water and sediment [4,32]. To date, Malaysia is still lacking studies to address the exposure of perfluorinated compounds in food. Therefore, findings from the present study provide an initial assessment on PFC contamination in food (poultry eggs) from several locations across Malaysia. Based on the results obtained, several recommendations can be made to improve the study outcome. First, the sample collections should focus on free-range chickens, since commercially produced chickens are less likely to be contaminated by perfluorinated compounds due to their habitat and feeding habits. The best location would be an area closely situated to industrial activities, which would provide a good representation of the pollution. Further studies should also include health risk assessments of PFC exposure from food consumption in local populations. This would require information on the average daily intake of the food, and reference values of average body weight in the population. In addition, the study should expand to other food sources and exposure pathways, as the consumption of other foods, such as fish, would be more significant, compared to the intake of eggs.

## 4. Materials and Methods

### 4.1. Chemicals

Native and mass-labelled perfluoroalkyl carboxylic acid (PFOA) and perfluoroalkyl sulfonate (PFOS) solution/mixture (2.0 µg/mL in methanol) were obtained from Wellington Laboratories Inc., Guelph, Ontario, Canada. Acetonitrile, methanol and ammonium acetate were bought from Fisher Scientific, Fair Lawn, NH, USA. ELGA Purelab Option ultra-pure water system was supplied by ELGA Lab Water, High Wycombe, Buckinghamshire, UK.

### 4.2. Instrumentation

**Figure 5:**
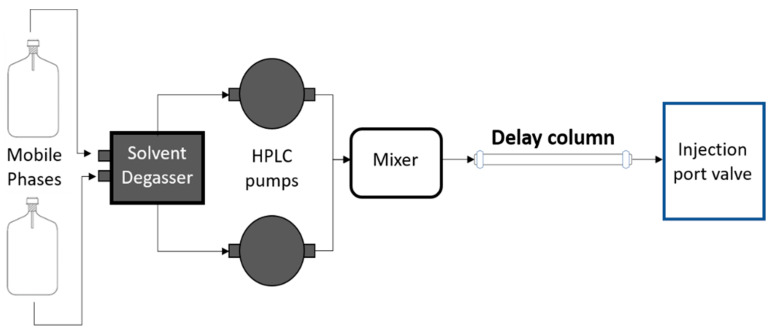
Column configuration.

The liquid chromatographic analysis was performed using Shimadzu Prominence ultra-fast liquid chromatography system (UFLC) (Shimadzu Corp, Kyoto, Japan). Separation was accomplished using the analytical column of Phenomenex Gemini NX-C18 (150 mm length X 2.0 mm internal diameter, particle size 5 µm) (Phenomenex, Torrance, CA, USA). A delay column (Figure 5) was installed in between the mixer and sample injector, in order to separate impurities, which may have existed in the chromatographic system. The mobile phase consisted of 2 mM ammonium acetate in water (mobile phase A) and methanol (mobile phase B), delivered at a flow rate of 0.35 mL/min. The gradient program was initially set at 20% B, and then ramped up to 98% B over 6 min, followed by an isocratic hold at 98% B until 9.00 min. At 9.01 min, the gradient was returned to 20% B and held further until 11.00 min. The total run time for each injection was 11 min, the sample injection volume was set at 5.0 µL, and the column temperature was maintained at 40 °C.

The mass spectral analysis was performed by using AB SCIEX QTRAP 5500 triple quadrupole mass spectrometer (Foster City, CA, USA), operating in electron spray ionization (ESI) negative ion mode. PFOS and PFOA MS/MS were detected by direct infusion of the reference standards (native and mass-labelled) dissolved in methanol, at the concentration of 1 µg/mL. Optimum mass spectrometric parameters, such as declustering potential (DP), collision energy (CE), entrance potential (EP), and collision cell exit potential (CXP), were obtained for both PFOS and PFOA. Detection and quantification of both analytes were performed using multiple reaction monitoring (MRM) transitions, whereby, parent and daughter ions for each compound can be simultaneously measured. The ion intensity for both precursor and product ion (*m*/*z*) were monitored, and the most intense product ion of each compound was selected for quantification.

### 4.3. Sample Collection and Preparation

The egg samples (*n* = 47) used in the study were collected from several different regions across Malaysia from January 2019 to February 2019. The commercially produced chicken eggs, bought mainly from the supermarket, consisted of different brands and grades (*n* = 30). The home-produced chicken eggs (*n* = 10) were obtained from local residents of Penang (*n* = 3), Kedah (1), Pahang (*n* = 3), Terengganu (*n* = 1), Kelantan (*n* = 1), and Selangor (*n* = 1); who reared chicken domestically. The duck (*n* = 3) and quail (*n* = 4) eggs samples were collected from both commercial and home-produced sources. Following collection, the eggs were taken to the laboratory for sample preparation. The eggshells were first cleaned with tap water, to remove external contaminants, followed by methanol, and further cleaned with ultra-pure water. The yolk of each egg was carefully separated from the white part, pooled in a polypropylene bottle, and homogenized. Each sample consisted of three to five yolks (unless fewer provided). The egg yolk samples were stored in −20 °C freezer, prior to analysis.

### 4.4. Preparation of Standards and Quality Control Materials

Master stock solution of target analytes was prepared in 100 ng/mL by diluting the native and mass-labelled standard mixtures (2 µg/mL), using 100% methanol. All of the working standard concentrations (PFOS: 0.5, 1, 2, 5, 10, 15 and 20 ng/mL; PFOA: 0.1, 0.2, 0.5, 1, 2, 5, 10, 15 and 20 ng/mL) and quality controls (0.75, 7.5 and 12.5 ng/mL) were prepared fresh by successive dilutions, and spiked into blank yolk samples before extraction.

### 4.5. Sample Extraction

Extraction of PFCs from egg yolk samples were carried out using a simple and rapid protein precipitation extraction. Approximately 1 g of egg yolk was fortified with 100 µL of mass-labeled PFCAs and PFSAs solution/mixture (50 ng/mL), as internal standards (IS). After spiking, 3 mL of acetonitrile was added to the yolk homogenates as extraction solvent. The mixture was vortexed for 1 min, followed by shaking at 200 rpm for 1 h. Subsequently, the extract was centrifuged for 15 min at 3500 rpm. The supernatant was collected and transferred into a new, clean polypropylene tube. The collected extract was evaporated until dry under a gentle stream of nitrogen gas. Following this, the dry residue was dissolved in 1 mL of methanol, vortexed, and filtered using a 0.2 µm syringe filter. The final solution was transferred into an autosampler vial, for analysis by LC-MS/MS.

### 4.6. Method Validation

The performance and consistency of the analytical procedures were validated based on FDA Bioanalytical Method Validation [33]. During the method validation, blank egg yolk samples were used throughout the analysis to obtain validation parameters, such as selectivity, calibration curve, sensitivity, accuracy and precision (intra and inter-assay), recovery, and stability.

#### 4.6.1. Selectivity

Four representative blank yolk samples of different sources were analyzed to evaluate the selectivity of the analytical method. In order to ensure high selectivity, the response of the biological blank must be lesser than the response of LLOQ standards, and retention time of both PFOS and PFOA in the standards and samples were compared at a tolerance of ±2.5%.

#### 4.6.2. Calibration Curve and Sensitivity

The calibration curve was evaluated by plotting the peak area ratio of the analyte to the internal standard versus the analyte concentration of spiked samples at seven different levels for PFOS, and nine different levels for PFOA. The evaluation was based on coefficient of determination (r^2^), with acceptable criteria of r^2^ > 0.99. The weighting factor used was 1/x, in which x is the concentration of PFCs and Y is the ratio of the chromatographic peak areas. LOD was determined from analyte of known low concentration, in which the response of signal to noise (S/N) ratio, is at least equivalent to 3. LLOQ, which is defined as the lowest concentration of an analyte, which can be reliably quantified, was determined from the analyte signal with an S/N ratio that was, at least, equivalent to 10.

#### 4.6.3. Accuracy and Precision

Accuracy and precision were measured by analyzing spiked egg yolk matrix at three QC concentration levels (low, medium, high), with five replicates each for three consecutive days. The accuracy of the method was expressed by the percentage of deviation between nominal and calculated concentrations, while the precision was expressed as coefficient of variation, CV (%).

#### 4.6.4. Recovery

Evaluation of absolute recovery was performed by comparing the analytical results of extracted QC samples, with corresponding extracts of blanks spiked with the analyte post-extraction.

#### 4.6.5. Stability

The analyte stability in the given matrix was evaluated through assessment of autosampler (Foster City, CA, USA) stability. This was done by keeping the QC samples in autosampler at 15 °C for 24 h.

### 4.7. Statistical Analysis

Statistical analysis was performed using IBM SPSS version 24 (Chicago, IL, USA). Appropriate statistical methods were used in order to evaluate the data. *p*-value of <0.05 were considered statistically significant.

## 5. Conclusions

A fast and reliable LC-MS/MS method in quantifying the concentration of PFOS and PFOA in the yolk of the egg samples was successfully achieved. In comparison to other methods, this technique has proven to be economical, less laborious, and sensitive for the determination of PFOS and PFOA in egg yolk samples in Malaysia. The present study provides initial data on the level of perfluorinated compounds (PFOS and PFOA) in egg samples in Malaysia, and future biomonitoring activities should be extended towards other food sources in the country. Further studies should also emphasize on the improvement of analytical methods to deal with certain drawbacks, such as matrix effects. It is highly recommended to replace the current chromatographic technique to sustainable chromatography by using green solvents in the future.

## Figures and Tables

**Table 1:** Intra- and inter-day accuracy and precision for PFOS and PFOA (mean ± SD).

Analyte	Concentration (ng g^−1^)	Intra-Assay (*n* = 5)			Inter-Assay (*n* = 15)		
		Observed Concentration (ng g^−1^)	Accuracy	RSD (% CV)	Observed Concentration (ng g^−1^)	Accuracy	RSD (% CV)
PFOS	0.50	0.54 ± 0.03	108	5.01	0.54 ± 0.03	108	6.35
	0.75	0.81 ± 0.02	108	2.03	0.81 ± 0.05	108	6.05
	7.50	6.85 ± 0.12	91.3	1.77	7.28 ± 0.50	97.1	6.91
	12.5	11.5 ± 0.28	92.3	2.42	11.8 ± 0.40	94.3	3.38
PFOA	0.10	0.11 ± 0.01	110	6.79	0.11 ± 0.01	110	6.25
	0.75	0.67 ± 0.01	89.3	1.82	0.69 ± 0.03	92.0	5.06
	7.50	7.38 ± 0.82	98.4	11.1	8.93 ± 0.68	101	4.08
	12.5	12.3 ± 0.51	98.4	4.19	12.4 ± 0.51	99.1	4.08

LLOQ = PFOS: 0.5 ng g^−1^; PFOA: 0.1 ng g^−1^, RSD = Relative standard deviation, CV = Coefficient of variation.

**Table 2:** Stability of PFOS and PFOA in egg yolk samples.

Analyte	QC Concentration (ng g^−1^)	Time	Mean Measured Concentration, ng g^−1^ (*n* = 5)	Accuracy (%)	RSD (% CV)
PFOS	0.75	0 hr	0.81 ± 0.02	109	2.03
		12 hr	0.80 ± 0.07	106	9.29
		24 hr	0.83 ± 0.04	111	5.13
	12.5	0 hr	11.5 ± 0.28	92.3	2.42
		12 hr	11.8 ± 0.42	94.2	3.57
		24 hr	12.1 ± 0.36	96.5	3.02
PFOA	0.75	0 hr	0.67 ± 0.01	89.7	1.82
		12 hr	0.73 ± 0.06	97.8	8.13
		24 hr	0.70 ± 0.03	93.0	4.82
	12.5	0 hr	12.3 ± 0.51	98.4	4.19
		12 hr	12.2 ± 0.57	97.6	4.67
		24 hr	12.7 ± 0.40	101	3.14

**Table 3:** PFOS and PFOA in egg yolk samples (*n* = 47).

Type	Source	Number of Yolk (n)	Concentration (ng g^−1^ ww)	
			PFOS	PFOA
**Chicken Eggs** **(Home Produced)**				
	1	3	0.64	<0.10 ^a^
	2	3	<0.50	<0.10
	3	3	0.50	<0.10 ^a^
	4	3	<0.50 ^a^	<0.10 ^a^
	5	3	1.01	<0.10
	6	3	0.53	<0.10 ^a^
	7	3	<0.50 ^a^	<0.10 ^a^
	8	3	<0.50 ^a^	<0.10
	9	3	0.52	<0.10
	10	3	<0.50	<0.10
Duck eggs				
	1	2	<0.50 ^a^	<0.10
	2	2	<0.50 ^a^	<0.10
	3	2	<0.50 ^a^	<0.10 ^a^
Quail eggs				
	1	5	<0.50 ^a^	<0.10
	2	5	0.69	<0.10
	3	5	<0.50 ^a^	<0.10 ^a^
	4	5	<0.50 ^a^	<0.10 ^a^

^a^ Concentration value lies between lower limit of detection (LOD) and LLOQ; LOD = PFOS: 0.1 ng g^−1^; PFOA: 0.02 ng g^−1^; LLOQ = PFOS: 0.5 ng g^−1^, PFOA: 0.1 ng g^−1^.
